# Progressive Realisation of Universal Health Coverage in Low- and Middle-Income Countries: Beyond the "Best Buys"

**DOI:** 10.34172/ijhpm.2020.245

**Published:** 2021-02-17

**Authors:** Melanie Y. Bertram, Jeremy A. Lauer, Karin Stenberg, Ambinintsoa H. Ralaidovy, Tessa Tan-Torres Edejer

**Affiliations:** ^1^Health Systems Governance and Financing, World Health Organization (WHO), Geneva, Switzerland.; ^2^Science Division, World Health Organization (WHO), Geneva, Switzerland.

**Keywords:** Cost-Effectiveness, Economic Evaluation, Efficiency, Universal Health Coverage

## Abstract

**Background:** World Health Organization Choosing Interventions that are Cost-Effective (WHO CHOICE) has been a programme of the WHO for 20 years. In this latest update, we present for the first time a cross-programme analysis of the comparative cost-effectiveness of 479 intervention scenarios across 20 disease programmes and risk factors.

**Methods:** This analysis follows the standard WHO CHOICE approach to generalized cost-effectiveness analysis applied to two regions, Eastern sub-Saharan Africa and Southeast Asia. The scope of the analysis is all interventions included in programme specific WHO CHOICE analyses, using WHO treatment guidelines for major disease areas as the foundation. Costs are measured in 2010 international dollars, and benefits modelled beginning in 2010, or the nearest year for which validated data was available, both for a period of 100 years.

**Results:** Across both regions included in the analysis, interventions span multiple orders of magnitude in terms of cost-effectiveness ratios. A health benefit package optimized through a value for money lens incorporates interventions responding to all of the main drivers of disease burden. Interventions delivered through first level clinical and non-clinical services represent the majority of the high impact cost-effective interventions.

**Conclusion:** Cost-effectiveness is one important criterion when selecting health interventions for benefit packages to progress towards universal health coverage (UHC), but it is not the only criterion and all calculations should be adapted to the local context. To support country decision-makers, WHO CHOICE has developed a downloadable tool to support the development of data for this criterion.

## Background

Value for money, efficiency and impact are fundamental considerations for strategic investment in health at national and global level.^1^ With finance no longer considered the greatest barrier to achieving better health outcomes, making strategic choices at country level becomes more important than ever.^2,3^

As a decision-making criterion within health, cost-effectiveness analysis helps countries and donors to ensure that they get the best value for money possible from the resources being expended.^4^ Although the sole focus of results presented in this paper, cost-effectiveness analysis is only one part of the priority setting process, not a singular criterion, and needs to be considered along with other concerns like equity, gender and human rights, and the need to avoid financial impoverishment on the part of those who seek care.^5,6^ Countries undertaking priority setting and decision-making processes within the health sector increasingly focus on three main steps: collecting data, undertaking a deliberative dialogue, and the political decision-making process.^7^ In many countries with advanced institutional arrangements, this priority setting and decision-making process in enshrined within a health technology assessment mechanism, with cost-effectiveness as a key criterion contributing to intervention selection processes.^8,9^

Cost-effectiveness analysis is a critical criterion for developing health benefit packages that will move countries towards universal health coverage (UHC) and the Sustainable Development Goals (SDGs). World Health Organization Choosing Interventions that are Cost-Effective (WHO CHOICE) published a series on cost-effectiveness analysis to support health systems in achieving the millennium development goals in 2005,^10-15^ and expanded the evaluation to non-communicable diseases in a 2012 series of papers.^16-18^ Since then, however, technical and operational knowledge about interventions has evolved. Disease epidemiology too has changed, as have populations.^19,20^ Costs have shifted, with changing economic conditions as well as developments in production, distribution and delivery models, necessitating an update of the WHO CHOICE analyses.^21-25^

The use of generalized cost-effectiveness analysis in WHO CHOICE provides some distinct advantages in developing generic UHC benefit packages.^26^ Because all interventions are compared to the same “null” scenario, intervention packages can be developed for any strata of interest building on the most cost-effective intervention option as the start point. For example, benefit packages can be developed for each individual health programme,^27^ for sub-sectors such as HIV, tuberculosis and malaria^28^ across the whole health system, or for each service delivery level (eg, population-based interventions, primary healthcare [PHC], referral level services).^29^

 Cross-sectoral packages are arguably the most pertinent for UHC. All decisions about which interventions to include in a package come with an opportunity cost, meaning that some alternative intervention cannot be funded and the health benefit of the non-funded intervention will not be realized. Given that health funding is generally pooled, so all health services are vying for a share of the same pot of money, the opportunity costs of making a decision are necessarily cross-sectoral.

 In this paper, we present stylized health benefit packages using an allocative efficiency lens. This means that we use cost-effectiveness as a sole criterion, with the expectation that any country use of these benefit packages would entail adaptation of these data in the context of local data on epidemiology and costs, along with health sector priorities and additional criteria that reflect local values.

We present first a system wide benefit package for the Southeast Asian global burden of disease region, followed by benefit packages for population level services and primary level clinical services separately for the Eastern sub-Saharan Africa global burden of disease region (see methods paper^26^ for information on selected regions). This separation by service delivery level may be useful in country settings where the health system is not functioning well enough at present to support the rapid implementation of additional services or coverage scale up of existing services which require inpatient or outpatient service delivery capacity, but where population health and outreach services can be delivered immediately.

## Key Messages

**Implications for policy makers**

- Cost-effectiveness analysis can be a useful tool for policy-makers to ensure public funds are being used in the most efficient and effective way possible. All countries should be encouraged to identify their own health benefit package for universal health coverage (UHC). World Health Organization Choosing Interventions that are Cost-Effective (WHO CHOICE) provides a methodological approach and toolkit which can support policy decision-makers in identifying and quantifying the trade-offs inherent in the decision-making process.
- For all disease areas included in the analysis, cost-effective interventions that fall on the expansion path have been identified.
- Service packages will differ based upon epidemiological profile, health expenditure and local values. All countries should develop a process through which interventions to be funded are identified in a transparent, fair manner.

**Implications for public**

Health benefits which the public is entitled to through universal health coverage (UHC) packages should be clearly communicated to the public. Along with this, the methodology for choosing which interventions are funded should be explicit, transparent and fair. The public has a vested interest in ensuring public funds are use efficiently, and World Health Organization Choosing Interventions that are Cost-Effective (WHO CHOICE) can be one tool used by policy-makers to strengthen the data aspects of their decision-making processes.

**What was already known on this topic?**

Cost-effectiveness analysis has been considered as a core criterion for health benefit package selection for many years, and was identified as one of the three core values in the WHO report of Making Fair Choices on the path to UHC. WHO CHOICE has been developing tools to support country level cost-effectiveness for 20 years.

**What does this paper add to the topic?**

For the first time, a cross-disease package has been developed using a common method for cost-effectiveness. This paper shows how cost-effectiveness can be used to support the development of an optimized health benefit package to support progress to UHC. The paper also presents a tool kit for country decision-makers to use to contextualize these analyses to their own setting.

## Methods

For this WHO CHOICE sectoral analysis, we take the cost-effectiveness ratios for 479 interventions across 20 disease/risk factor groups that have been calculated using the common generalized cost-effectiveness methodology of WHO CHOICE. Methodological assumptions and results by disease are published in separate papers in this series. The full WHO CHOICE generalized cost-effectiveness methodology has been published separately,^10,21,26^ all updates and relevant methods for this series are described in full in a separate paper within this series^26^ and disease specific adaptations outlined in the appropriate papers^27,28,30^ These interventions reflect either single interventions, interventions analyzed at varying coverage levels, or small bundles of interventions for which there is a clinical reason to deliver as a package. The scope of the analysis is all interventions included in programme specific WHO CHOICE analyses, using WHO treatment guidelines for major disease areas as the foundation. Costs are measured in 2010 international dollars using a health system perspective, and benefits modelled beginning in 2010, or the nearest baseline for which validated data is available, both for a period of 100 years. In this paper, we present the results with 3% discounting of costs and 0% discounting of health benefits only, although results in earlier papers in this series have presented both 0% and 3% discounting of health benefits. For this WHO CHOICE series, cost-effectiveness ratios have been calculated for the Eastern sub-Saharan Africa and Southeast Asian global burden of disease regions. For country specific applications it is anticipated that the WHO CHOICE toolkit is used to develop country specific estimates to ensure validity of cost-effectiveness ratios.

 In order to develop expansion pathways our first step is to rank all interventions in order of average cost-effectiveness ratio for each region. The intervention with the lowest average cost-effectiveness ratio is the first point on the expansion path in moving away from the null (zero cost, zero health benefit) scenario. The second step is to calculate the incremental cost-effectiveness ratio of adding the next most cost-effective intervention to the package. This can be either a new intervention, or an increase in coverage of the same intervention. This involves calculation of the incremental health benefits – which are in effect lower than implementing an individual intervention, as some health benefit has already been obtained with intervention one – and incremental costs which should include economies of scale thus not be entirely additive between interventions. The only exception to this is when the next addition to the package is from a different health programme. At this stage we make the assumption that costs and benefits are additive to the previous point on the expansion path.

 We identify at each step interventions which are dominated, ie, have lower health benefits and a higher cost than the intervention being added to the package. Dominated interventions are not able to be included in the package. In some instances interventions sit very close together in terms of cost-effectiveness, so in a real-world scenario may still remain valid additions to the benefit package, but for this mathematically optimized path are excluded. We continue this process until all interventions are either included in the package, or excluded as a potential option.

 To create expansion paths across levels of care – for example for public health interventions and PHC interventions – the same approach is used within sub-groups of interventions delivered at the same platform level. For each intervention, we use delivery guidelines or technical expert advice to determine the most common delivery platform. We assume that even when developing benefit packages by platform, programme support is still needed across each discrete disease programme area. This aligns with the authors experience of the structure of programmes in countries. An alternative approach could be to assume that programmatic support is provided for public health intervention programmes, or primary health intervention programmes. This impact of such a decision would be to increase the economies of scale which exist within a programme, thus leading to diminishing costs of adding each new intervention to the package. Thus, the approach we use in this paper is the more conservative option.

 With some limited exceptions (for example cardiovascular disease and diabetes, which are modelled simultaneously) this approach ignores co-morbidities. Although all models used in the analysis link to the same demographic projection, incidence rates are modelled independently. This is a limitation of the approach, which stems from a lack data at the country level on the correlations between the diseases to incorporate in these global models.

## Results

**Figure 1:**
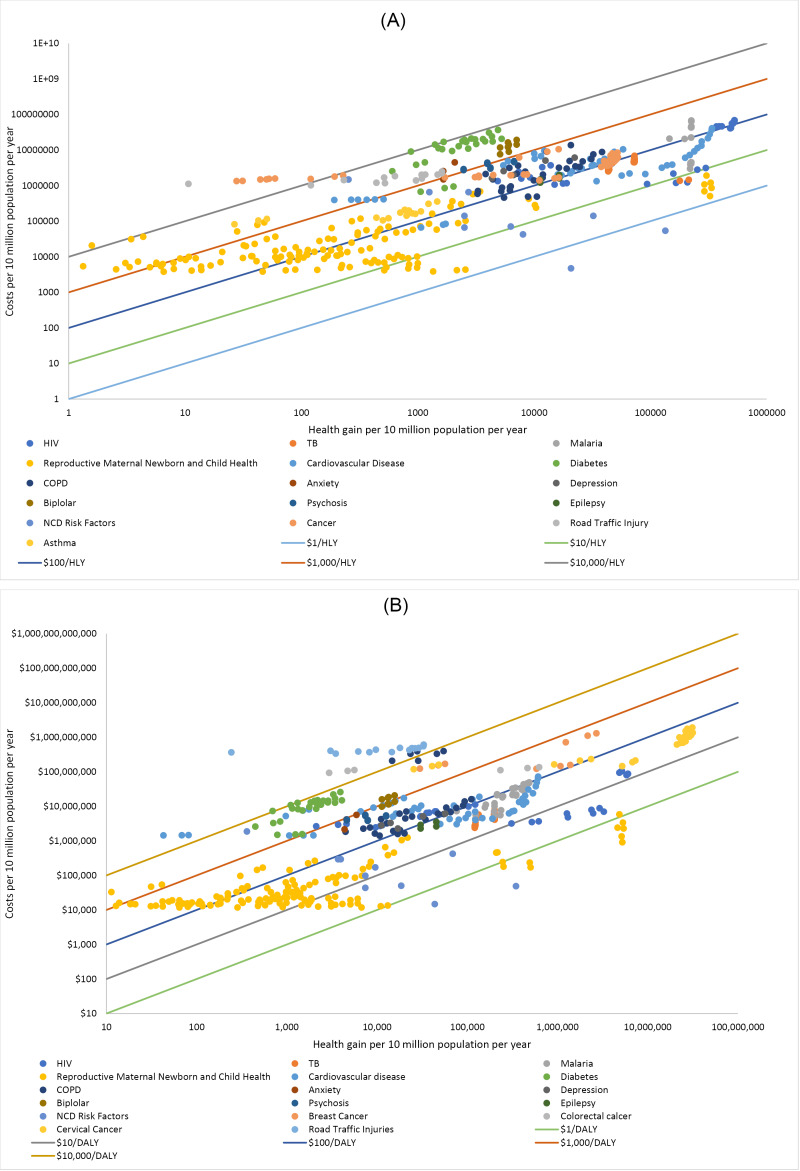
(A) Cost-Effectiveness Ratios for 479 Interventions in South East Asia Region on a Log-Log Scale. **(B) **Cost-Effectiveness Ratios for 483 Interventions in Eastern Sub-Saharan Africa Region on a Log-Log Scale. Abbreviations: COPD, chronic obstructive pulmonary disease; NCD, Chronic obstructive pulmonary disease; DALY, disability-adjusted life-year; HLY, healthy life years; TB, tuberculosis.

In both regions, the spectrum of interventions evaluated span multiple orders of magnitude in terms of cost-effectiveness ratios. Figure 1A displays the cost-effectiveness ratios for all 479 interventions for South East Asia on a log-log scale graph, also knowns as an “isoquant graph,” where each of the diagonal lines represent an order of magnitude difference on cost-effectiveness ratio. In addition, interventions towards the lower end of the vertical axis represent lower cost alternatives, whereas towards the right hand side of the horizontal axis interventions have higher health benefits, but with the same resulting cost-effectiveness ratio within the isoquant bands. Figure 1B displays the 483 interventions for Eastern sub-Saharan Africa in the same manner. The difference in intervention numbers is due to different numbers of malaria interventions plotted for vivax versus falciparum malaria strains. Interventions falling under the $1 per healthy life year gained line are limited to population level policy interventions, specifically tobacco taxation and reduction in sodium levels in manufactured foods. Many disease areas begin to have interventions available at the $10 per healthy life year gained and $100 per healthy life year gained cost-effectiveness bands, with the few exceptions being diabetes care, lung diseases and road traffic injury prevention. There is no intention within this analysis to identify a supply side threshold for funding of health services, the use of the diagonal bands showing order-of-magnitude differences in cost-effectiveness ratio is intended only as a useful visual guide.

 Essentially, all disease areas have good buys and less good buys, and this graph is indicative of the spread of cost-effectiveness ratios of interventions present in most countries health benefit packages. Presenting the results in this way shows the sometimes very small differences between the cost-effectiveness of interventions, and the difficulties policy-makers often face in making marginal decisions between these interventions.

**Figure 2:**
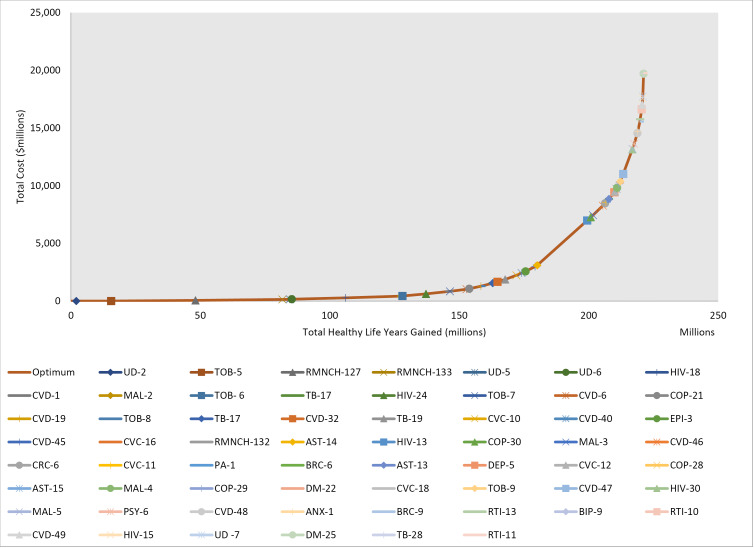
Hypothetical Optimal Health Benefit Package in South East Asia Region (See Supplementary file 1 for intervention descriptions). Abbreviations: UD, unhealthy diet; CVD, cardiovascular disease; TOB, tobacco; EPI, epilepsy; TB, tuberculosis; RMNCH, reproductive, maternal, newborn and child health; CRC, colorectal cancer; CVC, cervical cancer; PA, physical activity; BRC, breast cancer; DEP, depression; AST, asthma; COP, chronic obstructive pulmonary disease; DM, diabetes mellitus; MAL, malaria; PSY, psychosis; ANX, anxiety; RTI, road traffic injuries; BIP, bipolar disorder.

Developing the mathematically optimal pathway across all disease areas again indicates a mix of diseases would have interventions falling into a health benefit package (Figure 2). This particular example at the South East Asian regional level identifies a package of 60 interventions beginning with an average cost-effectiveness ratio of $0.23/healthy life year gained for sodium reduction policy as the first point on the expansion pathway, through to a final point with a near-vertical incremental health benefit which is the addition of legislation and enforcement of helmet use by bicyclists at an incremental cost-effectiveness ratio of $23692. It is important to note that as these are stylized regional level service packages, and not directly implementable in any specific country, no budget constraint has been enforced on the package. At the individual country level, a budget constraint could be used to identify the cut-off point for the package.

This package (see Supplementary file 1) could be delivered in the long run for $19.80 per capita per year, but this per capita amount represents the long-run costs in a health system with appropriate infrastructure, human resources and supply chain to support delivery of the interventions. It does not give a good indication of the immediate financial needs for health systems investments and health workforce development, nor does it account for interventions which may be slightly less cost-effective but still deliver excellent value for money and have additional compelling reasons for including in a benefit package.

In the context of progressing towards UHC and meeting the SDGs by 2030, many countries are focusing on PHC as the channel through which to catalyse SDG progress. For the eastern sub-Saharan Africa region, we analysed a hypothetical package of PHC services that could be delivered, first by generating league tables across all diseases of the average cost-effectiveness ratio of interventions delivered at the population level, through community-based and outreach programmes and through first level clinical services, following the baseline definition used in the guide posts for financial investment in PHC.^31^ This package has eliminated any overlaps caused by interventions evaluated at multiple coverage levels or in overlapping bundles.

Presenting the list of interventions as in Table is a different approach from the expansion path (Figure 2) in that it allows the inclusion in the package of interventions with similar levels of cost-effectiveness without explicitly excluding those “dominated” interventions. This listing of interventions differs from the expansion path displayed for South East Asia, in that it is not mathematically optimized but rather presents all those interventions included in our analyses which fall into order of magnitude cost-effectiveness bands, and could all be considered for implementation.

**Table:** Summary of Interventions Delivered Through Different Delivery Platforms and Their Average Cost-Effectiveness Ratios, Eastern Sub Saharan Africa, East

**Population Level Interventions**	
<$10 per healthy life year gained	**ACER**
Implement multicomponent salt reduction strategies in community settings including schools, workplaces and hospitals	$0.3
Complete elimination of industrial trans fats	$0.4
Raise taxes tobacco	$3
Mass media communication designed to increase demand and improve use of condoms, and condom provision	$3
Enforce advertising bans tobacco	$6
Set target levels for the amount of salt in food and implement strategies to promote reformulation	$6
$10 -$100 per healthy life year gained	
Offer to help quit (brief intervention) tobacco	$13
Protect - smoke free policies	$18
Adopt interpretive front-of-pack nutrient labelling systems	$74
Warning labels tobacco	$79
Smoking cessation, 95% coverage	$81
**Community-Based Services**	
<$10 per healthy life year gained	
Community-based management of pneumonia	$3
Education for female sex workers to prevent HIV	$6
Vitamin A supplementation (0-4 years)	$7
$10 -$100 per healthy life year gained	
Home visits for clean postnatal practices	$12
Infant and young child feeding	$12
Community-based newborn and child care	$14
Management of diarrhea through oral rehydration solution and zinc	$22
$101 -$1000 per healthy life year gained	
People who inject drugs community outreach and peer education to prevent HIV	$249
Education for men who have sex with men to prevent HIV	$673
**First Level Clinical Services**	
<$10 per healthy life year gained	
Neonatal resuscitation	$1
Voluntary medical male circumcision for HIV prevention	$2
Facility based management of pneumonia	$3
Facility based management of neonatal infection (sepsis/pneumonia) with injectable (and oral) antibiotics	$8
$10-$100 per healthy life year gained	
Measles vaccine	$11
IMCI sick child	$12
Routine EPI	$16
Management of children with severe acute malnutrition	$17
Pentavalent (DPT + Hep B + Hib)	$22
Tetanus toxoid vaccination	$23
Combination Therapy for patients with total CVD risk > 30%	$23
Clean cord care (clean birth practices)	$24
H. influenzae b	$25
Syphilis detection and treatment	$25
Balanced energy-protein supplementation to pregnant women with insecure food availability	$28
HPV vaccination (2 doses) for preventing cervical cancer	$29
Promotion of breastfeeding	$29
Expanding CVD prevention to those at >20% risk	$29
Treating those with high blood pressure but low absolute CVD risk	$31
Treating those with high cholesterol but low absolute CVD risk	$36
Promotion of complementary feeding	$37
Rotavirus vaccine	$44
Intermittent presumptive treatment of malaria	$54
Routine EPI + rotavirus, pneumococcal	$64
Management of pre-eclampsia (mild and severe)	$85
Management of suspected uncomplicated cases + management of severe cases of malaria	$94
Hypertensive disease case management	$95
Malaria RTS,S vaccine in addition to previous malaria interventions	$95
$101-$1000 per healthy life year gained	
Malaria diagnostics (additional to previous malaria interventions)	$100
Inhaled salbutamol for COPD	$105
Post event treatment as secondary prevention for CVD	$108
Daily iron and folic acid supplementation in pregnant women	$111
Antibiotics for treatment of dysentery	$113
Aspiring for stroke prevention	$119
Safe abortion services	$144
Ipratropium inhaler for COPD	$336
Folic acid supplementation	$356
$1001 + per healthy life year gained	
Calcium supplementation in pregnant women for the prevention and management of pre-eclampsia/eclampsia	$1311
Standard glycemic control for diabetes	$5747
Stepwise approach to asthma treatment	$8717
Intensive glycemic control for diabetes	$9648
**Referral Level Interventions**	
<$10 per healthy life year gained	
Skilled delivery plus management of complications plus family planning	$0.4
Case management of severe neonatal infection (sepsis/pneumonia) with full supportive care	$4
$10-$100 per healthy life year gained	
Case management of newborn complications at referral level	$14
Kangaroo mother care	$20
Management of severe cases of malaria	$24
Skilled assistance for normal delivery	$30
Skilled assistance plus management of complications during delivery	$57
Full supportive care for premature babies	$63
Management of maternal sepsis	$93
Diagnosis and treatment of cervical cancer stages I and II (using surgery, radiotherapy and chemotherapy as needed)	$100
$101-$1000 per healthy life year gained	
Pharmaceutical treatment of stroke and ischemic heart disease event	$108
Intensive psychosocial treatment and anti-depressant medication for recurrent moderate-severe cases of depression on a maintenance basis	$108
Diagnosis and treatment of breast cancer stages I and II (with surgery, radiotherapy and chemotherapy and hormone therapy as needed)	$113
Oral prednisolone for COPD exacerbation	$114
Intensive psychosocial treatment and anti-depressant medication for recurrent moderate-severe cases of depression on an episodic basis	$160
Antibiotics for preterm premature rupture of membranes	$184
Post abortion case management	$198
Diagnosis and treatment of colorectal cancer stages I and II (with surgery, chemotherapy and radiotherapy as needed)	$217
Intensive psychosocial treatment and anti-depressant medication for first-episode moderate-severe cases of depression	$236
Basic psychosocial treatment and anti-depressant medication for first-episode moderate-severe cases of depression	$252
Antibiotics for COPD exacerbation	$290
Management of eclampsia with magnesium-sulphate	$294
Antipsychotic medication + intensive psychosocial treatment of psychosis (older drugs)	$405
Antipsychotic medication + intensive psychosocial treatment of psychosis (newer drugs)	$435
Screening with mammography (once in 2 years for the age group 50 to 69 years) linked with timely diagnosis and treatment	$485
Antipsychotic medication + basic psychosocial treatment of psychosis (older drugs)	$716
Antipsychotic medication + basic psychosocial treatment of psychosis (newer drugs)	$766
Mood-stabilizing medication + basic psychosocial treatment for bipolar disorder (older drugs)	$849
Mood-stabilizing medication + intensive psychosocial treatment for bipolar disorder (older drugs)	$867
Basic psychosocial and anti-depressant drug treatment for moderate-severe cases of anxiety disorder	$918
Intensive psychosocial and anti-depressant drug treatment for moderate-severe cases of anxiety disorder	$955
$1001+ per healthy life year gained	
Neuropathy screening and preventive foot care for patients with diabetes	$1059
Ectopic pregnancy case management	$1156
Basic palliative care for breast cancer: home-based and hospital care with multi-disciplinary team and access to opiates and essential supportive medicines	$3009
Basic palliative care for cervical cancer: home-based and hospital care with multi-disciplinary team and access to opiates and essential supportive medicines	$3316
Retinopathy screening + photocoagulation for patients with diabetes	$4335
Basic palliative care for colorectal cancer: home-based and hospital care with multi-disciplinary team and access to opiates and essential supportive medicines	$20117

Abbreviations: ACER, average cost-effectiveness ratio; IMCI, integrated management of childhood illness; CVD, cardiovascular disease; HPV, human papillomavirus; COPD, chronic obstructive pulmonary disease; EPI, expanded Programme on Immunization; Hep B, hepatitis b; Hib, haemophilus influenzae type b; DPT, diphtheria, pertussis (whooping cough), and tetanus.

The package of 66 interventions at first level of clinical services is largely low cost and highly cost-effective. Population level interventions could be delivered for as little as $1.20 per person per year on average, community-based interventions for $1.00 per capita and first level clinical services $23.75 per capita. Again, this does not account for the immediate financial needs to strengthen health systems, which are reflected in the global price tag for PHC.^31^

**Figure 3:**
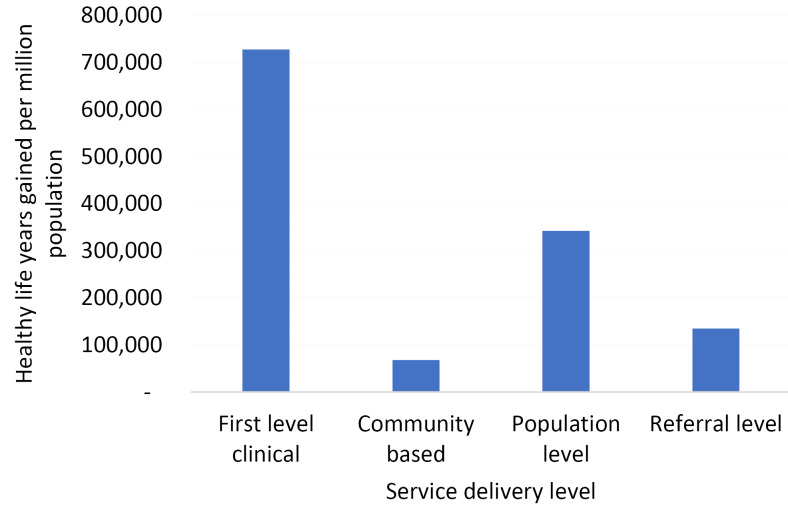
Health Gains by Service Delivery Level Due to Packages Shown in Table.

At the referral level, defined here as everything outside of the PHC definition,^31^ interventions tend to have less impressive cost-effectiveness ratios, but as the population in need is lower the overall cost for the 41 interventions presented is $20.30 per capita. These interventions do not gain the magnitude of health benefit that those interventions delivered through first-level services are able to do, with the primary levels of services being the drivers of health gain (Figure 3). Importantly, population level services which do not rely on health system strengthening, and are often considered common goods for health,^3^ can achieve large health benefits and be rapidly implemented.

## Discussion

 The results aim to show stylized benefit packages developed based on cost-effectiveness ratios. The results show that for most disease areas, it is possible to identify good value for money options, and there are likely other intervention options which represent a less efficient use of resources. This means that for every disease programme, there are interventions that would be part of a health benefit package developed based on value for money considerations. Overall, the WHO CHOICE Toolkit enables the development of an optimal expansion path across diseases, and when compared to the current country health benefit package this can support the identification of inefficiencies in resource allocation.

The use of a consistent methodology across all disease areas provides the WHO CHOICE analysis with some advantages over the use of cost-effectiveness data from different sources which may experience issues in comparability of methodology.^32^ League tables that are truly comparable and avoid the known pitfalls of comparing the outcomes of CEA which have different methods, can support decision-making processes by providing consistent information for decision-making. In addition, by generating the null scenario, an optimized pathway across multiple disease areas can be developed.

 Once the position of allocative efficiency has been estimated, it is possible to engage in strategic planning ie, bringing priority setting concerns explicitly into decision-making processes. By looking at the optimal package through a value for money lens, compared to the proposed strategy of the country, and given how non-optimized health systems often are, it will usually be possible to identify ‘quick wins’ and ‘low hanging fruit.’ These would be changes in the current set of activities that are politically feasible, affordable, technically possible, and which also improve health system efficiency, or interventions that would benefit from targeted price negotiation to reduce costs and improve the value-for-money. Yet even in the absence of such easy wins, it will be possible to identify opportunities over the short term (usually 5-year) planning horizon where politically feasible, affordable and technically possible changes can be made. Opportunities for disinvestment or for increased investment can be identified. Without an explicit priority setting focus, however, such opportunities will be systematically missed.

Bottlenecks in health system resources such as personnel and facilities, or weak capacity in priority setting, strategic planning and purchasing, or monitoring and evaluation, can become barriers to further progress once an initial level of investment has been made. Linking health priorities and decision-making to considerations around feasibility of implementation is crucial to creating accountability within health sector planning.^29^

 At present, the WHO CHOICE tool kit is limited by the number of health impact models present, which do not yet correspond to all health conditions which should be considered as part of UHC Packages. This limitation should not be taken as an implication that missing disease areas are not important, but rather that they are at present beyond the technical limits of the models available to us. New impact modules are added over time and intervention information is updated as WHO recommendations change.

 Many of the 20+ impact modules in the toolkit have been developed independently, meaning that independence of interventions across diseases is assumed. This may overestimate in some instances the health benefits achieved by interventions, however equally costs may be overestimated as there may be potential for economies of scale being achieved through integrated treatment.

Inevitably the package presented will be compared to the Disease Control Priorities Essential UHC package.^33^ Whilst there are significant overlaps in the results presented, methodological differences do drive some of the findings. For example, all analyses presented by WHO are primary cost-effectiveness analyses, using a common methodology. As already noted, 20 diseases and/or risk factors are included in the analysis. For Disease Control Priorities, cost-effectiveness analyses are drawn from the existing literature, meaning a broader number of health conditions is able to be included, however the comparability across studies is reduced. WHO CHOICE has not attempted to select priority interventions through the incorporation of additional criteria, based on the belief that this information is highly contextual to the individual country, and cannot be done at the global level.^1^Disease Control Priorities has attempted to address additional criteria in order to develop a final proposed package. Both approaches can be used as part of a comprehensive health benefit package development process at the country level, as has been demonstrated in Ethiopia.

Although the results presented in this paper are calculated at the regional level, the paper also presents the concepts behind an underlying global public good, a free to download and use toolkit that enables country users to develop their own estimates of cost-effectiveness in their setting for all the interventions presented in this series of papers. Both cost-effectiveness analysis and strategic planning and scenario costing should be reviewed regularly, as prices and the countries epidemiological profile change with time, leading to changes in the optimal mix of interventions and optimal implementation strategies. WHO has made the full WHO CHOICE tool kit available at https://www.avenirhealth.org/software-onehealth.php.

Ideally country decision-makers, or their designated alternatives, would be the users of such a tool kit, enabling all countries to develop the data they need to support benefit package decision-making, leading to more efficient distribution of resources. This data can be used in conjunction with information on other criteria in order to select health benefit packages which are fit for purpose for achieving UHC. Common criteria for decision-making include cost-effectiveness analysis, budget impact, priority to the worst off, financial risk protection, feasibility and others.^6,35^

 This paper demonstrates how cost-effectiveness ratios across multiple disease areas can be used to inform resource allocation decisions, and can support processes for health benefit package selections. Countries should be encouraged to use data relevant to the local context within a fair, transparent decision-making process to ensure progress towards UHC.

## Acknowledgements

 The authors wish to acknowledge the contribution of Rory Watts from the Department of Health Systems Governance and Financing at WHO and Dan Chisholm from the WHO Regional Office for Europe for contributing to underlying analyses that contribute to this paper, and for contributions to discussions on methodology.

## Ethical issues

 No ethical approval was sought as this is a secondary data analysis.

## Competing interests

 Authors declare that they have no competing interests.

## Authors’ contributions

 MYB led the cross disease analysis included in this paper, led the analysis on NCDs and drafted this manuscript. JAL provided technical input into the method for sectoral analysis and edited the manuscript. KS led the analysis on RMNCH and edited this manuscript. AHR led the analysis for HIV, TB, malaria, cancer and RTI included in this analysis. TTTE oversaw the development of new methodology and has edited the manuscript.

## Disclaimer

 MYB, JAL, KS, AHR and TTTE are staff members of the WHO. The views expressed in this paper are solely the responsibility of the named authors and do not necessarily reflect the decisions or stated policy of the WHO or its Member States.

## Authors’ affiliations

^1^Health Systems Governance and Financing, World Health Organization (WHO), Geneva, Switzerland. ^2^Science Division, World Health Organization (WHO), Geneva, Switzerland.

## Supplementary files

Supplementary file 1. Expansion Path for South East Asia.
