# Supplementary material for: Progressive Realisation of Universal Health Coverage in Low- and Middle-Income Countries: Beyond the "Best Buys"
Source: Int J Health Policy Manag. 2021 Feb 17;10(11):697–705. doi: 10.34172/ijhpm.2020.245 (PMC9278372; doi:10.34172/ijhpm.2020.245)
Supplement: Supplementary file 1 — Expansion Path for South East Asia. [file ijhpm-10-697-s001.pdf]

Supplementary file 1. Expansion Path for South East Asia

**Intervention name**	**ICER**	**Cumulative Cost Per Capita**
Set target levels for the amount of salt in food and implement strategies to promote reformulation	$0.2	$0.00
Raise Taxes on Tobacco	$0.4	$0.01
Skilled delivery + management of complications_50	$2	$0.06
Newborn complications at referral level_80	$3	$0.14
Interpretive front of pack labelling for foods	$4	$0.16
Sodium reduction package	$5	$0.16
Education for Female Sex Workers to prevent HIV	$6	$0.28
Management of severe cases	$7	$0.43
Enforce advertising bans on tobacco	$11	$0.44
Mass Media communication designed to increase demand and improve use of Condoms, and condom provision	$21	$0.63
Prevention: Comb. Tx for total CVD risk > 30%, 50% coverage	$23	$0.84
Tobacco warning labels	$26	$0.84
Prevention: Comb. Tx for total CVD risk > 20%, 50% coverage	$28	$1.02
Smoking cessation, 95% coverage	$47	$1.07
Comb. Tx for total CVD risk > 20%, 80% coverage	$50	$1.29
Tobacco smoke free policies	$56	$1.31
Comb. Tx for total CVD risk > 20%, 95% coverage	$60	$1.67
Treatment (FLD +SLD) +Detection (Smear+ X-ray+ Culture) +Drug susceptibility testing & ART Prioritization for TB cases	$69	$1.87
HPV vaccination (2 doses) for preventing cervical cancer & Prevention of cervical cancer through screening with HPV test: women 30-49 years every 3 years linked with timely treatment of pre-cancerous lesions	$85	$2.24
prevention - 20% risk plus 160mmHG	$93	$2.43

Antiepileptic Medication + Basic Psychosocial treatment of epilepsy (older drugs)	$97	$2.58
prevention- 20% risk + 6mmol/L+160mmHg	$112	$2.99
HPV vaccination (2 doses) for preventing cervical cancer & Prevention of cervical cancer through screening with VIA: women 30-49 years every 3 years linked with timely treatment of pre-cancerous lesions	$127	$3.02
48_P6. Skilled delivery + management of complications + family planning	$154	$3.09
Theophylline + High dose inhaled beclometasone +SABA, 95% coverage	$169	$3.11
Antibiotics plus prednisolone plus oxygen, 95% coverage	$203	$7.28
prevention- 20% risk + 6mmol/L+140 mmHg	$218	$8.27
Diagnosis & treatment of colorectal cancer stages I and II (with surgery, chemotherapy and radiotherapy as needed)	$238	$8.48
Brief Intervention - Physical Activity	$240	$8.64
Diagnosis & treatment of breast cancer stages I and II (with surgery, radiotherapy and chemotherapy and hormone therapy as needed)	$252	$8.84
High dose inhaled beclometasone +SABA, 95% coverage	$256	$8.85
Intensive psychosocial treatment and anti-depressant medication for recurrent moderate-severe cases of depression on a maintenance basis	$275	$9.43
Salbutamol plus theophylline, 95% coverage	$348	$9.71
Oral Prednisolone + Theophylline + High dose inhaled beclometasone +SABA, 95% coverage	$416	$9.73
Salbutamol plus theophylline plus inpratropium, 95% coverage	$447	$10.15
Neuropathy screening and preventive foot care, 95% coverage	$465	$10.24
Brief intervention for smoking cessation	$521	$10.38
Prevention package plus treat stroke	$559	$11.02
TASP: HIV testing services +Antiretroviral Therapy treatment as prevention for all adults, children and PMTCT Option B+	$603	$13.15
Management of severe cases & Insecticide-treated bed nets	$671	$13.19

Antipsychotic Medication + Intensive Psychosocial treatment of psychosis (older drugs)	$683	$13.63
Prevention package plus treat stroke plus treat ischemic heart disease	$761	$14.56
Basic psychosocial treatment for mild cases of anxiety disorder	$903	$14.70
Screening with mammography (once in 2 years for the age group 50 to 69 years) linked with timely diagnosis and treatment	$1,048	$15.55
Speed cameras & Drink-drive legislation + enforcement (breath-testing) & Legislation + enforcement of helmet use by motorcyclists	$1,381	$15.76
Mood-Stabilizing Medication + Basic Psychosocial treatment for bipolar disorder (older drugs)	$1,487	$16.61
Legislation + primary enforcement of seat belt use & Legislation + enforcement of helmet use by motorcyclists & Speed cameras & Drink-drive legislation + enforcement (breath-testing)	$1,552	$16.63
Prevention package plus treat stroke plus treat ischemic heart disease plus aspirin	$2,278	$17.02
CB3: TASP + Mass Media and Condom +Female Sex Workers +Injecting drug users + Men who have Sex with Men +Youth Focused Interventions +STI +Voluntary Medical Male Circumcision	$2,781	$17.58
Complete elimination of industrial trans fats	$5,849	$17.73
Standard GC plus neuropathy screening, 95% coverage	$6,109	$19.71
Treatment (FLD +SLD) +Detection (Smear+ X-ray+ Culture) +Drug susceptibility testing & Preventive therapy for children	$10,230	$19.73
Legislation + primary enforcement of seat belt use & Legislation + enforcement of helmet use by motorcyclists & Speed cameras & Drink-drive legislation + enforcement (breath-testing) & Legislation + enforcement of helmet use by bicyclists	$23,692	$19.76
